# Coronavirus Pneumonia: Outcomes and Characteristics of Patients in an Inner-City Area after 3 Months of Infection

**DOI:** 10.3390/jcm10153368

**Published:** 2021-07-29

**Authors:** Gilda Diaz-Fuentes, Gabriella Roa-Gomez, Olga Reyes, Ravish Singhal, Sindhaghatta Venkatram

**Affiliations:** Division of Pulmonary & Critical Care, BronxCare Health System, Bronx, NY 10457, USA; ggomez@bronxcare.org (G.R.-G.); oareyes@bronxcare.org (O.R.); rsinghal@bronxcare.org (R.S.); svenkatr@bronxcare.org (S.V.)

**Keywords:** chest radiography, COVID-19, pneumonia, post coronavirus pneumonia, post COVID

## Abstract

Background: The morbidity and long term pulmonary consequences of COVID-19 infection continue to unfold as we learn and follow survivors of this disease. We report radiological evolution and pulmonary function findings in those patients. Methods: This was a retrospective cohort study of adult patients referred to the post-acute COVID-19 pulmonary clinic after a diagnosis of COVID-19 pneumonia. The study period was after the initial peak of the pandemic in New York City, from June to December 2020. Results: 111 patients were included. The average interval time between COVID-19 pneumonia and initial clinic evaluation was 12 weeks. 48.2% of patients had moderate and 22.3% had severe infection. Dyspnea and cough was the most common respiratory symptoms post infection. Radiographic abnormalities improved in majority of patients with ground glass opacities been the common residual abnormal finding. Restrictive airway disease and decreased diffusion capacity were the most common findings in pulmonary function test. Conclusion: Our study suggests the needs for close and serial monitoring of functional and radiological abnormalities during the post COVID-19 period. Considering that many of the clinical-radiological and functional abnormalities are reversible, we suggest a “wait and watch“approach to avoid unnecessary invasive work up.

## 1. Introduction

A novel coronavirus, the severe acute respiratory syndrome coronavirus 2 (SARS-CoV-2), causing the coronavirus disease (COVID-19) emerged in December 2019 and triggered a global pandemic. The morbidity and long-term pulmonary consequences of COVID-19 infection are unfolding as we continue to learn and follow survivors of this disease.

The Centers for Disease Control and Prevention guidelines identify several risk factors for severe COVID-19 disease and complications, including older age, obesity, and several chronic lung conditions, like moderate to severe asthma, chronic obstructive airway disease (COPD), interstitial lung disease, cystic fibrosis, and pulmonary hypertension [1]. The acute pulmonary and extrapulmonary manifestation of COVID-19 infection has been well-described in the literature, and they can have long-term unknown consequences [2,3,4]. Studies of COVID-19 survivors suggest that some patients have persistent respiratory dysfunction, as well as persistent radiographic abnormalities that could last long after the acute infection subsides [5,6]. In a study by Carfi, 87.4% of patients were symptomatic after 2 months of infection [7]. Wu and colleagues followed patients after COVID-19 hospitalization, and persistent physiological and radiographic abnormalities were found in 24% of their patients [8].

Long-COVID, or post-COVID syndrome, has been defined by the National Institute for Health and Care Excellence (NICE) as “signs and symptoms that develop during or after an infection consistent with COVID-19 which continue for more than 12 weeks and are not explained by an alternative diagnosis”. The long-term pulmonary and systemic sequelae is of increased concern.

We conducted a single-center study in an inner-city ambulatory post-acute COVID-19 pulmonary clinic in New York City. The aim was to evaluate outcomes of patients after COVID-19 pneumonia. We evaluated the radiological and pulmonary function findings in those patients.

## 2. Patients and Methods

### 2.1. Study Design and Patients

This was a retrospective cohort study conducted at an inner-city community teaching hospital, serving the South and Central Bronx population. Our population is composed mainly of Hispanic and African American individuals with multiple comorbidities.

The pulmonary clinic setting includes an on-site pulmonary physician and a respiratory therapist. Spirometry and chest radiography (CXR) can be performed during the same clinic visit. Referral to pulmonary rehabilitation or other required services is readily available.

We included adult patients referred to the post-acute COVID-19 pulmonary clinic after a diagnosis of COVID-19 pneumonia. All individuals had a documented positive nasal swab COVID-19 test result. Inclusion criteria included: (a) patients with a diagnosis of COVID-19 pneumonia, (b) patients with at least two clinic visits post-COVID-19 infection; (c) face-to-face clinic visit, and (d) more than 2 months after initial COVID-19 infection. Patients seen via tele-health were excluded.

The study period was after the initial peak of the pandemic in New York City, from June to December 2020.

Ethics approval: The study protocol was approved by the Institutional Review Board (approval number 10082009) and followed the amended Declaration of Helsinki. The need for informed consent was waived, as this was a retrospective study.

### 2.2. Data Abstraction

All data were retrospectively extracted from medical records, including the patients’ demographic, clinical, radiological, and pulmonary function test (PFT) details. Imaging was evaluated by a thoracic radiologist.

A review of an available echocardiogram to assess the presence of pulmonary hypertension was done. A cut-off of 36 mmHg for systolic pulmonary artery pressure was chosen. Greiner et al. showed that Doppler echocardiography had good diagnostic sensitivity (87%), specificity (79%), and accuracy (85%) for a systolic pulmonary artery pressure cutoff value of 36 mmHg [9].

## 3. Results

During the study period, 111 patients met the inclusion criteria. The mean age of the group was 60 ± 13.9 years, and the majority of patients were Hispanic and female. Significant comorbidities included hypertension, obesity, diabetes mellitus, asthma, and chronic obstructive airway disease (COPD); cigarette smoking, either active or former, was found in 36% of our patients (Table 1).

The average interval time between COVID-19 pneumonia and initial clinic evaluation was 12 weeks, with a range of 8–16 weeks.

The World Health Organization (WHO) ordinal scale criteria were used to classify the severity of COVID-19 infection. The majority, or 48.2% of our patients had moderate disease, followed by 22.3% with severe infection [10], as shown in Table 2.

### 3.1. Symptoms

Dyspnea, followed by chronic dry or mild productive cough, was reported in 55.4% and 42.9% of patients, respectively, on their initial presentation to the clinic. Symptoms suggestive of broncho-constriction, like chest tightness and wheezing, were reported in 8.1% of the patients. There were 18 patients who were discharged from the hospital with supplemental oxygen; only six of them required supplemental oxygen after 4 months of follow-up, as shown in Table 3.

### 3.2. Chest Imaging

Chest imaging performed during the acute infection, either CXR or chest CT, was available for review in 59 (53.1%) of the patients. Comparison of chest imaging obtained during the acute and post-acute infection revealed a trend for improvement or resolution of radiographic abnormalities.

Imaging was obtained as per the treating pulmonologist. Of the 111 patients, 106 (95.4%) had CXR, and 39 (35.1%) had chest CT imaging obtained post-acute infection. Persistent CXR and chest CT abnormalities were seen in 33.9% and 27.9% of patients, respectively. Discrepancies between rates of abnormalities between CXR and chest CT are likely due to time lag, as CTs were performed at least 3–6 weeks after post-infection CXR. The most common persistent radiologic finding in chest CT was ground glass opacification in 24 (77.4%) out of the 31 patients with abnormal CT, shown in Table 4.

Other rare findings were mediastinal lymphadenopathy, cavity and nodular lesions. Chest CT post-COVID-19 was performed at an average of 16 weeks post-acute infection.

### 3.3. Pulmonary Function Test

We had complete pulmonary function test with spirometry with and without bronchodilators, single-breath diffusion capacity, and nitrogen washout in 63 (56.8%) of patients. Eight patients were not able to perform the test due to dyspnea and cough. The main indication for the PFT was evaluation of dyspnea or persistent cough.

Normal PFTs were seen in 18 (28.6%) patients, restrictive airway disease in 33 (52.4%), and decreased diffusion capacity in 39 (61.9%) patients. Mixed obstructive and restrictive findings were seen in 6.3% of patients (Table 5).

We correlated the severity of COVID-19 infection and pulmonary function test abnormalities, and found no correlation. Contrary to our expectations, there were patients with mild/moderate disease, that is, according to the WHO clinical progression scale of 1–6, who had severe decreased diffusion capacity, and other patients with more severe disease with mild impairment.

The most common radiological abnormality post-acute COVID-19 infection was ground glass opacification in chest CTs. We had 39 patients who underwent a post-COVID-19 chest CT, and ground glass abnormalities were found in 24; however, only 14 patients had PFTs available.

Of those 14 PFTs, eight showed restrictive disease, three obstructive airway diseases, all had decreased diffusion, and three PFTs were completely normal.

There were 48 (43.2%) patients with available echocardiograms performed during COVID-19 infection, where 13 (27%) had a sPAP > 36 mmHg. In those patients with pulmonary hypertension by echocardiogram, the diffusion capacity was decreased in 12 (92%) of them (seven mild, four moderate, and one severe decreased diffusion).

One patient died during the follow-up period (etiology unknown).

## 4. Discussion

We report the characteristic features of high-risk patients after COVID-19 infection in an inner-city hospital post-discharge with residual respiratory symptoms. Our study confirms findings reported by others of the persistent respiratory symptoms, functional and radiographic abnormalities after several months of acute COVID-19 pneumonia. Dyspnea was reported in 55%, and chronic cough in 42.9% of our patients; chest tightness and wheezing was reported by 8% and 4.5%, respectively. The latter two symptoms have not been mentioned in the literature as part of chronic symptoms post-COVID-19 infection and they could be related to underlying obstructive airway disease. In the Long-COVID syndrome, coughing and dyspnea are prominent, lasting for several months, with coughing been present in 15% to 42% of patients [7,11,12,13]. The supplemental oxygen requirement for post-acute COVID-19 infection ranges from no requirements to up to 50% of patients being discharged on oxygen, and this wide variation is likely due to the time-frame of the study, as most patients will not require oxygen after 4 to 6 months post-infection [14,15]. Factors reported to be predictive of post-COVID syndrome are female sex, severity of acute infection, and the presence of respiratory comorbid conditions [12,13,16]. In contrast to other studies on Long-COVID respiratory symptoms, our population had a higher incidence of obesity and other comorbidities, and patients were also older.

Current evidence suggests wide variations in the prevalence and incidence of post-COVID-19 syndrome. Sample size, the population studied, and different follow-up periods possibly account for some of these variations.

Persistent radiological and functional abnormalities have been reported in a growing number of patients post-acute COVID-19 infection, including patients with few or no comorbid conditions and patients with mild or moderate infection [17,18].

The incidence and resolutions of chest abnormalities range widely in the literature, and it is likely related to the time-frame of the follow-up. A post-COVID-19 evaluation of 384 hospitalized patients in London showed that 38% of them had persistent CXR findings after an average of 54 days [11]. A prospective French study of 478 survivors of COVID-19 requiring hospitalization showed that after four months, 16% still had dyspnea; chest CT abnormalities were seen in 63% of 171 patients with fibrotic changes in 19%, and the worst in patients that had acute respiratory distress syndrome [13]. Some other smaller studies reported similar fibrotic radiological findings [19,20]. A Chinese study with serial chest CTs looking at 51 patients post-COVID infection showed that after 4 weeks of infection, a significant number of patients had improvement or resolution of radiological abnormalities [21]. A prospective longitudinal study by Wu and colleagues following 83 patients for 12 months after hospitalization for COVID-19 infection showed that 24% had residual abnormalities in chest CTs [8]. The most commonly described persistent imaging abnormalities include ground glass opacification and reticulation, as found in our study.

Data looking at the pulmonary function test in this group of patients have a wide variation; evaluations range from evaluation of oxygenation, where some studies report pulmonary function tests with or without diffusion capacity, and a few report the performance of a 6 min walk test. In our study, 61.9% of patients had decreased diffusion capacity. Our cohort included patients with underlying pulmonary conditions, like obstructive airway disease, which could make interpretation of obstructive pattern findings difficult.

Decreased diffusion capacity of the lung has been consistently reported and seems to correlate with more abnormal radiological findings [8,9,10,11,13,17]. Similarly to discrepancies found with chest imaging, findings in PFTs vary, and we suspect one of the main variables is timing of the PFT as related to the acute COVID-19 infection, as well as to the severity of lung involvement and prior undiagnosed lung conditions. In general, diffusion abnormalities in PFT are the most common abnormality found associated in some cases with restrictive patterns; obstructive patterns are less common [6,8,21]. In a series of 28 patients post-severe COVID-19 infection requiring hospitalization and followed within 39 days, 61.5% had normal lung function and 26.9% had reduced diffusion capacity; 24 of those patients were on mechanical ventilation [22]. Another study of 124 patients from the Netherlands evaluated after 3 months of infection found that 42% had abnormal diffusion capacity, and this correlated with the severity of illness and extension of lung parenchymal involvement [19]. Serial monitoring of PFTs has shown that with time, abnormalities tend to improve [8].

In our study, 10% of CT scans post-infection revealed fibrotic lesions and reticulations; 61.5%, ground glass opacification; and 71% of PFTs showed some type of abnormality. This indicates a high burden of post-COVID-19 respiratory disease. Considering the millions of people affected by COVID-19, the long-term burden of patients with persistent respiratory symptoms, and radiological and functional abnormalities will be substantial. This suggests an urgent need to establish post-COVID-19 syndrome clinics, and integrated care with multidisciplinary teams to address specific patients’ needs. A blueprint to create those settings was outlined by Lutchmansingh and colleagues [23].

Teams caring for those patients, especially pulmonologists, will be better served having a clear understanding of the epidemiology, clinical course, and characterization of the chest imaging and pulmonary function test. This will help to create some expert guidelines for clinical evaluation, follow-up, and resource allocation.

Our study has some strengths and limitations. The strengths are as follows: (a) we studied a group of patients still not well-characterized in the literature; (b) we selected only patients with post-COVID-19 pneumonia to better evaluate persistent respiratory symptoms; and (c) a mix of high-risk ethnic minorities in a medically under-served area was included, where these groups are usually under-represented in most studies. Limitations include the retrospective single-center nature of the study and a lack of available baseline pulmonary function tests prior to COVID-19 infection. PFTs were performed mainly in those patients with persistent dyspnea and not systematically in all patients with post-COVID-19 infection, meaning those patients with poor perception of dyspnea may have been missed. Lastly, chest imaging during the acute phase of the infection was not available for radiology review in close to 50% of patients with post-infection, and not all patients had chest CTs, either due to insurance limitations or because they were not ordered by the treating physician.

## 5. Conclusions

This study provided some answers, but also generated many more questions, regarding the long-term impact of COVID-19 infection in the respiratory system. This was a “real life” study in an underserved area with mixed ethnic groups, higher comorbidities, and many limitations with regard to care. We were able to understand the respiratory symptoms burden, radiological evolution, and impact of pulmonary function tests in survivors of post-acute COVID-19 pneumonia. Data generated by this study and others suggest the need for close and serial monitoring of clinical, functional, and radiological abnormalities. Given the high number of infected patients, and considering that pulmonary symptoms usually predominate, a sequel of COVID-19 would likely represent a major disease burden, especially for pulmonary specialists.

Systems need to be put in place to provide care to those patients and work with multidisciplinary teams to address specific needs, as nicely outlined by Lutchmansingh et al. [23].

Considering that many clinical–radiological and functional abnormalities are reversible, it would not be unreasonable to monitor those patients closely and wait several months prior to embarking on a more aggressive approach to avoid unnecessary invasive tests.

## Figures and Tables

**Table 1:** Clinical characteristics of patients with post-COVID-19 pneumonia.

Variable	*n* (%)
Age, yr ± standard deviation	60 ± 13.9
Gender, female	59 (53.1)
Race	
Black	22 (19.8)
Hispanic	62 (55.9)
White	2 (1.8)
Other	25 (22.5)
Body mass index, kg/m^2^	31.4 ± 8.2
<30	56 (50.5)
31–39	45 (40.5)
>40	10 (9.0)
Comorbidities	
Hypertension	67 (60.4)
Diabetes mellitus	34 (30.6)
Asthma	25 (22.5)
Chronic obstructive airway disease	12 (10.8)
Human immunodeficiency virus	7 (76.3)
Heart failure	8 (7.2)
Malignancy	5 (4.5)
Peripheral vascular disease	5 (4.5)
Obstructive sleep apnea	4 (3.6)
Active or Former Tobacco Smoking	40 (36.0)
Echocardiogram	48 (43.2)
Systolic Pulmonary artery pressure < 36 mmHg	35 (72.9)
Systolic Pulmonary artery pressure > 36 mmHg	13 (27)

**Table 2:** Severity of COVID-19 infection in our population based on the World Health Organization clinical progression scale.

Severity of COVID-19 Infection	Features	Scale	*n* = 111 (%)
Ambulatory, mild disease	Asymptomatic; viral RNA detected	1	0
	Symptomatic; independent	2	30 (27.0)
	Symptomatic; assistance needed	3	3 (2.7)
Hospitalized: moderate disease	Hospitalized; no oxygen therapy	4	18 (16.2)
	Hospitalized; oxygen by mask or nasal prongs	5	36 (32.4)
Hospitalized: severe diseases	Hospitalized; oxygen by non-invasive ventilation or high flow	6	15 (13.5)
	Intubation and mechanical ventilation, pO_2_/FiO_2_ ≥ 150 or SpO_2_/FiO_2_ ≥ 200	7	4 (3.6)
	Mechanical ventilation pO_2_/FiO_2_ < 150 (SpO_2_/FiO_2_ < 200) or vasopressors	8	2 (1.8)
	Mechanical ventilation pO_2_/FiO_2_ < 150 and vasopressors, dialysis, or extra corporeal membrane oxygenation	9	3 (2.7)
Dead	Dead	10	0

**Table 3:** Symptoms on presentation to the post-COVID-19 pulmonary clinic.

Symptoms	*n* = (%)
Asymptomatic	23 (20.7)
Shortness of breath	62 (55.9)
Cough	48 (42.3)
Chest pain	10 (9.0)
Chest tightness	9 (8.1)
Wheezing	5 (4.5)
Fatigue	6 (5.4)
Back pain	5 (4.5)
Anosmia	2 (1.8)
Disgeusia	1 (0.9)
Body aches	3 (2.7)
Joint pains	1 (0.9)

**Table 4:** Chest imaging findings in the 111 patients.

	Chest Radiography		Chest Computed Tomogram	
**Imaging Available for Review**	**During COVID ** ***n* = 58/111**	**Post-COVID** ***n* = 54/111**	**During COVID** ***n* = 28/111**	**Post-COVID** ***n* = 14/111**
**Radiologic findings**				
Normal	6 (10.3%)	35 (64.8%)	1 (3.6%)	2 (14.2%)
Unilateral infiltrates	12 (20.6%)	7 (12.9%)	2 (7.1%)	1 (7.1%)
Bilateral infiltrates	34 (58.6%)	11 (20.3%)	0	0
Bilateral dense consolidations	6 (10.3%)	0	2 (7.1%)	0
Ground glass opacification	0	0	19 (67.9%)	8 (57.1%)
Fibrotic lesions/reticulation	0	0	2 (7.1)	2 (14.2)
Other	0	1 (1.9%)	4 (14.3%)	3 (21.4%)
**Only Post Covid Imaging available for review**		**Post-COVID ** ***n* = 52/111**		**Post-COVID** ***n* = 25/111**
**Radiologic findings**				
Normal		35 (67.3%)		6 (24%)
Unilateral infiltrates		3 (5.8%)		1 (4%)
Bilateral infiltrates		6 (11.5%)		0
Bilateral dense consolidations		0		0
Ground glass opacification		0		16 (64%)
Fibrotic lesions/reticulation		0		2 (8)
Other		2 (1.9%)		2 (8%)

Others= Nodules, Pulmonary emboli, mediastinal lymphadenopathy, cavity, emphysema.

**Table 5:** Pulmonary function test findings in patients with post-COVID-19 infection.

Pulmonary Function Test	*n* = 63 (%)
**Normal**	18 (28.6)
**Obstructive pattern**	8 (12.7)
Mild	7
Moderate	0
Moderate Severe	0
Severe	1
Very Severe	0
Mixed Obstructive/Restrictive pattern	4 (6.3)
**Restrictive pattern**	33 (52.4)
Mild	13
Moderate	11
Severe	14
**Diffusion capacity**	
Normal	24 (38)
Mild	26 (41.3)
Moderate	12 (19)
Severe	1 (1.6)

## Data Availability

The data that support the findings of this study area available from the authors upon reasonable request.
